# Advocate the Voice of People with Dementia ‐ Design of Dementia Care Educational Package for the Practitioners with the 16 Principles of Proper Caring Attitude (DemenTitude®) in Hong Kong

**DOI:** 10.1002/alz.087773

**Published:** 2025-01-09

**Authors:** Kenny Chi Man Chui

**Affiliations:** ^1^ Hong Kong Social Worker Association, Hong Kong Hong Kong

## Abstract

Listening to the voices of people with dementia and understanding their perception of the surrounding world is one of the keys to providing person‐centred care in daily practice. The care not only focuses on the specific knowledge and skills but also the proper caring attitude towards dementia. Stigma is still severe in Hong Kong, and the implementation of dementia‐inclusive language is not practical due to the lack of education in this aspect. An educational package with 16 principles of proper caring attitude was designed regarding the local context and cultural adaption. Qualitative research with in‐depth interviews and participatory observations was conducted in the community care and residential care homes of Hong Kong. Following the theoretical framework blending interpretivism and the sociocultural perspective on dementia in Hong Kong, the 16 principles of proper caring attitude, called DemenTitude®, were formulated from the findings. The aims are to reduce the stigma of dementia among the caring staff and to raise awareness of dementia‐inclusive language.

The 16 principles were translated from Cantonese to English: (1) Unveiling limitless interpretations; (2) Listening more than criticise; (3) Memories fade, history endures; (4) Communication is all about interaction; (5) Trusting their feeling and perception; (6) The same stays the same; (7) Behavior speaks; (8) Beyond the matter‐of‐fact; (9) Process is the key for assessment; (10) Dignity through equality, treat as adults; (11) Meaningful engagements form life; (12) Mind your words; (13) The power of touch is better than words; (14) Person‐centred Care always as the root; (15) Quality and Quantity, both counts; (16) Care takes priority, “attitude” over “speed”. The evaluation of this educational training and care philosophy showed a significant improvement in the positive perception of dementia among the participants. Further development and promotion of “attitude” training should be needed.

